# Effects of Binaural Beat Music Integrated with Rhythmical Photic Stimulation on Anxiety Reduction among Healthy Daycare Center Staff

**DOI:** 10.1155/2024/5556702

**Published:** 2024-07-18

**Authors:** Cheng Liu, Shang-Yu Yang, Jiun-Yi Wang

**Affiliations:** ^1^Department of Physical Education, Huazhong University of Science and Technology, Wuhan 430074, China; ^2^Department of Healthcare Administration, College of Medical and Health Science, Asia University, Taichung 41354, Taiwan; ^3^Department of Medical Research, China Medical University Hospital, China Medical University, Taichung 404, Taiwan

## Abstract

This study investigated the efficacy of combining binaural beat music (BBM) with rhythmical photic stimulation at the *α* frequency in alleviating anxiety among daycare staff and explored its impacts on daycare staff with different anxiety levels. A quasi-experimental research design was adopted, which included three interventions: BBM, BBM integrated with rhythmical photic stimulation, and relaxation music (control group). Participants completed a questionnaire prior to the first intervention, which included personal demographic information and the Beck Anxiety Inventory. The effects of these interventions on anxiety relief among daycare staff were evaluated through heart rate variability (HRV), brain waves, and blood pressure before and after the interventions. Statistical analysis primarily employed the Friedman test to analyze the differences in changes in HRV, brain waves, and blood pressure before and after the interventions. A total of 40 individuals participated in this study (16 males and 24 females), with an average age of 31.73 ± 8.83 years. The results showed that, compared to BBM alone, BBM integrated with rhythmical photic stimulation significantly reduced the normalized low/high frequency (nLF/nHF) ratio in participants with moderate anxiety (*p* < 0.05). The results suggest that BBM integrated with rhythmical photic stimulation may serve as an intervention for the prevention and relief of anxiety by regulating an individual's autonomic nervous system. However, further research is required to confirm these findings.

## 1. Introduction

The global population is undergoing an aging process, and in response to the shortage of elderly care resources, services are offered through daycare centers to provide respite opportunities for family caregivers [1]. Consequently, the responsibility of caring for the elderly has shifted from households to the employees of these daycare centers [2]. Daycare staff contend with demanding, time-consuming, and often unacknowledged work that involves heavy labor. Their contributions are undervalued, and they receive little recognition for their efforts [3, 4]. Additionally, they must continuously address the needs and emotional responses of the elderly and their families while frequently facing physical assaults [5], sexual harassment [6], threats, and bullying [7]. During the COVID-19 pandemic, daycare staff were exposed to the risk of infection while continuing to provide professional care to the elderly, potentially worsening their anxiety symptoms [8, 9]. Anxiety not only causes distress for daycare staff themselves, but also leads to substantial medical expenses.

Music therapy has proven effective in alleviating symptoms of anxiety, with one promising approach being binaural beat music (BBM). In comparison to traditional relaxation music, BBM has demonstrated superior efficacy in enhancing emotional well-being [10], a potential benefit that may be reflected in brain wave patterns [11]. BBM's *α* frequency aligns with the inherent rhythm of the central nervous system, enabling it to synchronize with brain cells and activate *α* brain waves. Consequently, listening to BBM within the *α* frequency range (9–14 Hz) can stimulate the release of endorphins in the brain, fostering emotional stability, relaxation, and the relief of tension, anxiety, and other negative emotions [12]. Furthermore, listening to BBM may contribute to the regulation of emotional imbalances through enhancements to the autonomic nervous system [13]. Heart rate variability (HRV) is recognized as the most widely used method to assess the autonomic nervous system. In the study conducted by Sung et al. [13], individuals of an advanced age who listened to *α* (10 Hz) frequency BBM for five consecutive days experienced a notable reduction in the indicator of sympathetic nervous system activity, known as the low frequency (LF), along with a significant increase in the indicator of parasympathetic nervous system activity, known as the high frequency (HF). This implies that BBM has the potential to activate the parasympathetic nervous system and, in turn, alleviate anxiety levels among the elderly [10]. However, there remains a shortage of empirical research addressing whether BBM can ameliorate anxiety among daycare staff.

In recent years, there have been notable developments in the effectiveness of rhythmic photic stimulation in alleviating negative emotions [14, 15]. When the frequency of rhythmic photic stimulation closely aligns with the brain's occipital rhythm, neurons in the visual cortex can synchronize their activity under the influence of the stimulus, resulting in rhythmic entrainment [16]. Previous research [16] revealed that individuals with comorbid anxiety and depression experienced enhanced *α* brain wave activity and increased autonomic nervous system activity after undergoing rhythmic photic stimulation therapy, leading to an alleviation of their symptoms. Moreover, individuals with negative emotions tend to accumulate more amyloid-*β* in the brain than their optimistic counterparts, and elevated levels of amyloid-*β* are associated with an increased susceptibility to anxiety [17, 18]. A recent study conducted on mice [19] observed a significant reduction in amyloid-*β* levels in the brains of mice when subjected to simultaneous 40 Hz auditory and visual stimulation. This suggests that the combination of auditory stimulation (BBM) with visual stimulation (rhythmic photic stimulation) may have potential for the treatment and alleviation of anxiety, particularly involving the autonomic nervous system. Nonetheless, further empirical research is needed to establish its effectiveness conclusively.

In summary, both BBM and rhythmic photic stimulation may alleviate negative emotions. Prior to the main study, we conducted a pilot study with 10 healthy subjects aged 19–30 years. The findings revealed that BBM combined with rhythmical photic stimulation significantly reduced the normalized low/high frequency (nLF/nHF) ratio, indicating a positive effect on autonomic nervous system balance. The synergy of integrating BBM with rhythmic photic stimulation offers the possibility of enhancing and even multiplying their collective impact on easing anxiety symptoms. However, existing research in this field remains limited. Consequently, the primary objective of this study was to examine the influence of integrating *α*-frequency BBM with rhythmic photic stimulation in reducing anxiety and to assess its effectiveness among daycare staff with differing levels of anxiety.

## 2. Materials and Methods

### 2.1. Study Design and Participants

This study utilized an experimental crossover design conducted at a long-term care facility in central Taiwan. Participants were recruited between July 2021 and November 2021. Each participant underwent three different intervention groups: (1) BBM group (experimental group A); (2) BBM integrated with rhythmic photic stimulation group (experimental group B); and (3) relaxation music group (control group C). Six combinations of the sequence of these three interventions were denoted as ABC, ACB, BAC, BCA, CAB, and CBA. Participants were randomly allocated into one of the six sequences through a random draw method to minimize potential order effects.

**Figure 1:**
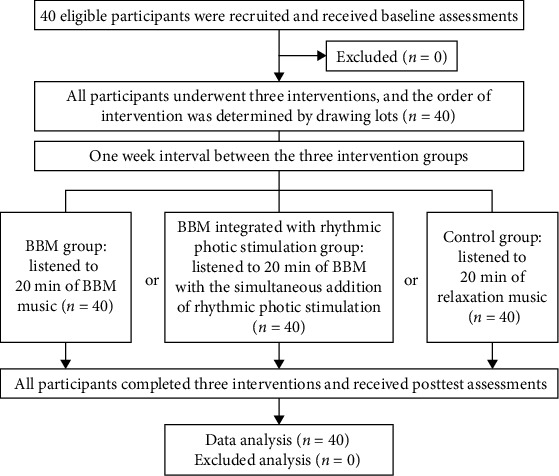
Research flowchart.

When completing a combination of three interventions, participants adhered to a minimum washout period of 1 week between interventions, as depicted in Figure 1. Inclusion criteria for participants were as follows: (1) aged 20 years or older, (2) employment duration of at least 6 months, and (3) no history of receiving treatment for anxiety or depression symptoms in the 3 months preceding the interventions and during the intervention period. Exclusion criteria included: (1) a documented history of mental illness, (2) recent exposure to highly stressful events within the past month (such as the loss of close relatives or friends), (3) a medical history of epilepsy, (4) ocular disorders, (5) experiences of dizziness (including Meniere's disease), and (6) hearing impairment.

In this study, the sample size was determined using G*⁣*^*∗*^Power with an effect size of 0.5, an *α* level of 0.05, and a power of 0.8, which estimated a minimum sample size of 35 participants. After research assistants explained the research purpose to potential participants, 40 individuals voluntarily enrolled in the study and provided written informed consent. The study was approved by the Research Ethics Committee of China Medical University Hospital (CMUH110-REC3-021).

### 2.2. Intervention

Participants completed a questionnaire prior to the first intervention, which included personal demographic information and the Beck Anxiety Inventory (BAI). Afterward, they rested for 10 min before undergoing pretesting involving HRV, brain wave measurements, and a blood pressure assessment. Subsequently, they received a 20-min intervention, and immediately following the intervention, posttesting, mirroring the pretest, was conducted. These interventions were scheduled between 9 : 00 AM and 12 : 00 PM in the lounge area of the long-term care facility, with only one participant present at a time.

For the BBM group, participants sat with their eyes closed, wearing stereo headphones. They listened to 20 min of relaxation music (nature sounds) infused with 10 Hz BBM. To ensure participants remained awake, a research assistant observed them 10 min after the intervention to confirm wakefulness.

**Figure 2:**
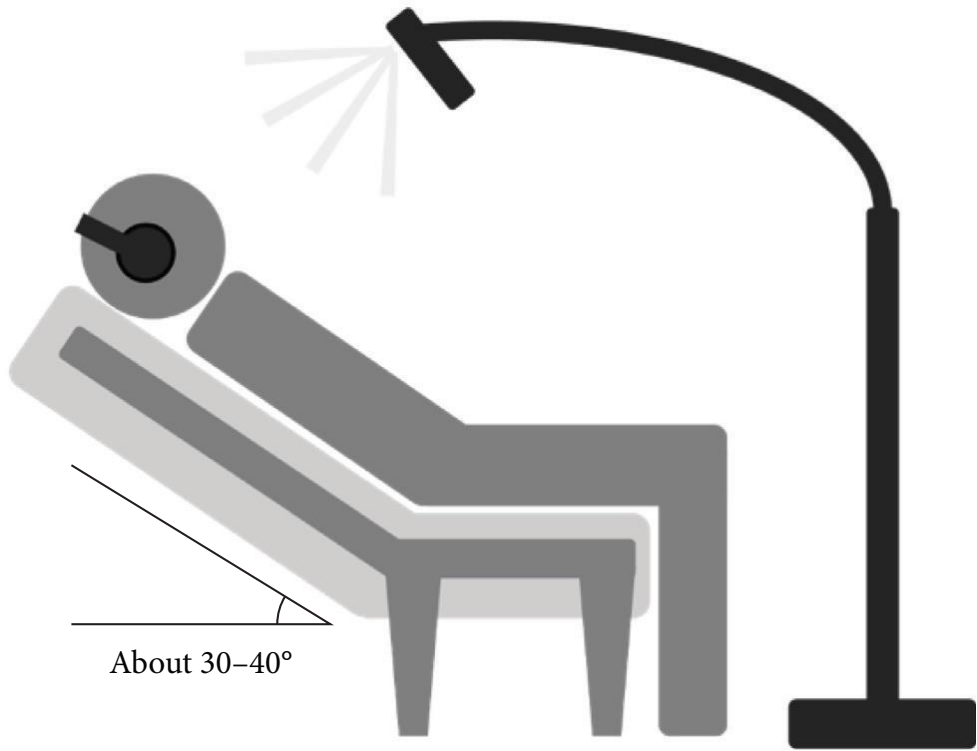
Schematic illustration of the intervention using BBM integrated with rhythmic photic stimulation.

For the BBM integrated with rhythmic photic stimulation group, the BBM listening procedure mirrored that of the BBM group, but with the simultaneous addition of rhythmic photic stimulation. The rhythmic photic stimulation also operated at a frequency of 10 Hz and was administered ~30 cm above the forehead, as illustrated in Figure 2.

For the relaxation music group, the intervention process was identical to that of the BBM group but without the inclusion of 10 Hz BBM.

### 2.3. Questionnaire

The collection of personal information encompassed gender, age, body mass index (BMI), education level, marital status, self-perceived health status, exercise habits (with sessions lasting 30 min or more), smoking behavior, alcohol consumption patterns, years of professional experience, and length of current employment.

The BAI served as a self-assessment tool to gauge the participants' anxiety levels based on their emotional experiences over the preceding 2 weeks. Comprising 21 questions, the BAI employed a four-point Likert scale, with scores ranging from 0 to 3, indicating the severity of distress: none (0 points), mild (1 point), moderate (2 points), and severe (3 points). Total scores ranged from 0 to 63, categorizing respondents into four anxiety levels: no anxiety (0–7 points), mild anxiety (8–15 points), moderate anxiety (16–25 points), and severe anxiety (26–63 points). Higher scores correlated with elevated anxiety levels [20]. The scale exhibited robust reliability and validity [21], with a Cronbach's *α* value of 0.90 in this study.

### 2.4. HRV

HRV measurements were conducted using the SA-3000P HRV analyzer (Medicore Co., Seoul, Korea). Participants underwent a 5-min HRV assessment while seated, from which five parameters were obtained, namely, (1) mean heart rate; (2) standard deviation of all RR intervals (SDNN), reflecting the physiological health of the autonomic nervous system, with higher values indicating better autonomic nervous system health; (3) normalized low frequency (nLF), indicating sympathetic nervous system activity, with higher values indicating better activity; (4) normalized high frequency (nHF), indicating parasympathetic nervous system activity, with higher values indicating better activity; and (5) nLF/nHF, an index reflecting the balance between the sympathetic and parasympathetic nervous systems. These parameters provided insights into changes in participants' sympathetic and parasympathetic nervous systems.

### 2.5. Brain Waves

The human brain generates continuous bioelectrical signals (brain waves) [22]. This study used the BrainLink Lite (Macrotellect Ltd., Shenzhen, China) to acquire brain signals [23]. The dry EEG electrodes in the elastic headband were placed close to the standard EEG locations F7 and Fp1 (bipolar channel). The third electrode is the ground (at Fpz location). The device was connected to a computer via bluetooth to record EEG output data per second. This data included *δ* (0.5–2.75 Hz), which represents the sleep stage; *θ* (3.5–6.75 Hz), which represents the day dreaming stage; low *α* (7.5–9.25 Hz) and high *α* (10–11.75 Hz), which represent the relaxation stage; low *β* (13–16.75 Hz) and high *β* (18–29.75 Hz), which represent the concentration stage; and low *γ* (31 Hz and up) and High *γ* (41–49.75 Hz), which represent the attention stage [24]. Additionally, the average values of the brain wave parameters (*δ*, *θ*, high *α*, high *β*, low *γ*, and high *γ*) were calculated for the 60 s before and after the intervention.

### 2.6. Sphygmomanometer

The OMRON HEM-7000 (OMRON HealthCare, Kyoto, Japan) blood pressure monitor was used to monitor the values of systolic and diastolic blood pressure before and after the intervention. A decrease in either the systolic or diastolic blood pressure value indicated a reduction in the participant's anxiety level.

### 2.7. Statistical Analysis

Data analysis was conducted using SPSS 25.0 for Mac (IBM Corp., Armonk, NY). First, descriptive statistics were employed to present participants' demographic characteristics. Participants were then categorized into three groups based on their BAI scores: no anxiety, mild anxiety, and moderate anxiety. Second, as the HRV, brain wave, and blood pressure values did not adhere to a normal distribution, the Wilcoxon signed rank test was employed to determine whether there were significant within-group changes in these values before and after the three different interventions. The change was defined as the value from posttest minus the corresponding value from pretest. They were presented using medians and quartiles. Third, the Friedman test was utilized to examine the between-group differences in changes among the three different interventions. When a significant result was observed from the Friedman test, post hoc pairwise comparisons were then conducted using the Wilcoxon signed rank test. The significance level was set at 0.05, except for the pairwise comparisons which was set at 0.017 according to Bonferroni correction.

## 3. Results

### 3.1. Personal Information

This study included a total of 40 participants, all of whom successfully completed the three interventions and required assessments.  Table 1 presents the participants' demographic information and BAI results. The participant group consisted of 16 males and 24 females, with an average age of 31.73 ± 8.83 years. None of the participants reported a smoking habit. BAI analysis revealed that the majority of participants exhibited mild anxiety (42.5%).

### 3.2. Changes in HRV, Brain Waves, and Blood Pressure before and after the Intervention

As depicted in Table 2, for the BBM group, (1) for participants with no anxiety, no significant differences in HRV, brain wave, or blood pressure parameters were observed between pre- and posttest; (2) for participants with mild anxiety, while HRV and blood pressure parameters showed no significant differences between pre- and posttest, there was a significant decrease in brain wave parameters, specifically in *θ*, low *α*, and high *α* (*p* < 0.01); and (3) for participants with moderate anxiety, no significant differences in HRV, brain wave, or blood pressure parameters were observed between pre- and posttest.

For the BBM integrated with rhythmic photic stimulation group, (1) for participants with no anxiety, there were no significant differences in HRV parameters between pre- and posttest. However, in brain wave parameters, there was a significant decrease in high *γ* (*p* < 0.05). Regarding blood pressure parameters, there was a significant reduction in diastolic blood pressure (*p* < 0.05). (2) For participants with mild anxiety, significant decreases were observed in mean heart rate and SDNN in HRV parameters between pre- and posttest (*p* < 0.05). In brain wave parameters, significant reductions were observed in *δ*, *θ*, low *α*, high *α*, low *β*, high *β*, low *γ*, and high *γ* (*p* < 0.01). No significant differences were noted in blood pressure parameters. (3) For participants with moderate anxiety, in HRV parameters, significant reductions were found in mean heart rate, nLF, and nLF/nHF (*p* < 0.05), while HF significantly increased (*p* < 0.05). There were no significant differences in brain wave parameters. In blood pressure parameters, both systolic and diastolic blood pressures significantly decreased (*p* < 0.05).

For the relaxation music group, (1) for participants with no anxiety, significant decreases were observed in nLF, nLF/nHF, and HF in HRV parameters between pre- and posttest (*p* < 0.05). No significant differences were noted in brain wave and blood pressure parameters. (2) For participants with mild anxiety, in HRV parameters, there were significant increases in nLF and nLF/nHF, along with a significant decrease in HF between pre- and posttest (*p* < 0.05). No significant differences were observed in brain wave parameters. In blood pressure parameters, systolic blood pressure showed a significant decrease (*p* < 0.01). (3) For participants with moderate anxiety, no significant differences were found in HRV and brain wave parameters between pre- and posttest. In blood pressure parameters, there was a significant decrease in systolic blood pressure (*p* < 0.05).

### 3.3. Differences in the Changes in HRV, Brain Wave, and Blood Pressure after the Intervention

Table 3 illustrates the variations in HRV, brain wave, and blood pressure changes before and after the intervention across the three groups, categorized by different anxiety levels. Only participants with moderate anxiety exhibited a noteworthy distinction in the nLF/nHF change within HRV (*p* < 0.05). Through post hoc analysis, further examination revealed that the BBM integrated with rhythmic photic stimulation group demonstrated a more substantial change compared to the BBM group, signifying that BBM combined with rhythmic photic stimulation exhibited superior performance in enhancing autonomic nervous system activity. Conversely, for participants with other anxiety levels, no notable differences were observed in the alteration of any measurement parameters among the three intervention groups.

## 4. Discussion

### 4.1. BBM Group

This study revealed a notable decrease in brain wave activity, particularly in the *θ*, low *α*, and high *α* bands, among participants with mild anxiety (Table 2). *α* waves are linked to relaxation and possess the capacity to synchronize with brain cells, contributing to anxiety alleviation [12]. Previous research [25] has shown that patients who listened to BBM incorporating *α* frequencies (e.g., 10 Hz) before surgery experienced a significant surge in *α* brain wave activity, resulting in reduced preoperative anxiety. Compared to prior studies, the differences in our study results may be attributed to stimulus adaptation. At times, the brain can adapt to sustained stimuli, leading to a gradual attenuation of the initial stimulus-induced changes in brain wave patterns [26]. This could potentially elucidate the declines observed in *θ*, low *α*, and high *α* brain wave activity. Furthermore, brain wave patterns are influenced by various factors, and participants may have concentrated on listening due to the novelty of experiencing BBM for the first time (as indicated by the rising trend in low *γ* activity). Therefore, it is advisable for future studies to offer participants a listening trial before the main body of the research.

Moreover, *θ* and *α* brain waves are traditionally associated with states of relaxation, meditation, and overall reduced arousal. If participants felt more relaxed while listening to BBM, this sensation could have contributed to the observed reductions in these particular brain wave frequencies. Nonetheless, responses to BBM can be highly individualized. In this study, participants with mild anxiety may have exhibited greater sensitivity to this form of stimulation, while those with no anxiety and moderate anxiety could have been less responsive. In summary, 10 Hz BBM may prove more efficacious for participants with mild anxiety.

### 4.2. BBM Integrated with Rhythmic Photic Stimulation Group

Among participants with no anxiety, significant reductions were observed in their brain wave activity (high *γ*) and diastolic blood pressure (Table 2). First, *γ* waves in the brain are high-frequency brain wave patterns typically associated with attention, perception, learning, and cognitive functions. When individuals enter a state of relaxation, the activity of *γ* waves in the brain may decrease [27]. Second, when individuals are physically and mentally relaxed, blood vessels tend to dilate, leading to a reduction in blood pressure [28]. This finding suggests that BBM combined with rhythmic photic stimulation may contribute to a certain relaxation effect among participants with no anxiety. However, it is possible that the intervention duration was too short to induce an increase in *α* waves. Future research may benefit from extending the intervention duration or frequency.

Two noteworthy findings were observed among participants with mild anxiety (Table 2). First, HRV showed significant decreases in mean heart rate and SDNN. Second, brain waves exhibited significant decreases in *δ*, *θ*, low *α*, high *α*, low *β*, high *β*, low *γ*, and high *γ*. The reduction in mean heart rate and SDNN likely reflects both physiological and psychological changes. While specific reasons warrant further investigation for confirmation, some plausible explanations can be proposed. In cases of mild anxiety, there may be an overactivity of the sympathetic nervous system, leading to an increased heart rate [29]. The intervention may have rebalanced the activity of the sympathetic and parasympathetic nervous systems, resulting in a lowered heart rate. SDNN is also influenced by autonomic nervous system activity and its decrease may indicate autonomic nervous system regulation, potentially contributing to anxiety reduction. Alternatively, previous studies [12, 30] found that BBM with an *α* frequency and rhythmic photic stimulation can increase *α* brain waves, leading to the alleviation of negative emotions in individuals. However, in this study, an overall decrease in brain waves was observed, which contrasts with previous findings. It is hypothesized that BBM combined with rhythmic photic stimulation intervention might have induced a state of profound relaxation, anxiety reduction, and distraction. The reduction in brain waves, particularly in the nLF (*δ* and *θ*) and nHF (high *α*, low *β*, high *β*, low *γ*, and high *γ*) ranges, may signify a less active or resting state of the participants' brains. Nevertheless, further research is required to gain a deeper understanding of the specific mechanisms underlying these results.

Among participants with moderate anxiety, this study revealed significant changes in HRV parameters, including decreases in nLF and nLF/nHF along with an increase in nHF (Table 2). Additionally, significant reductions in both systolic and diastolic blood pressure were observed (Table 2). These HRV findings are consistent with previous research, as both BBM [13] and rhythmic photic stimulation [16] have been shown to modulate HRV, resulting in anxiety relief. One study [13] reported a substantial overall improvement in HRV activity in elderly individuals who listened to BBM. The effectiveness of anxiety relief through rhythmic photic stimulation was also supported by HRV measurements [16]. Rhythmic photic stimulation induces *α* brain waves, leading to decreased nLF and increased nHF, which is beneficial for the recovery of patients with depression. Moreover, the blood pressure results are in line with previous research [31], where patients who listened to BBM before surgery experienced significant reductions in blood pressure and anxiety levels. These findings further validate the effectiveness of artificial light exposure in reducing blood pressure in healthy individuals [32]. Hence, the combination of BBM and rhythmic photic stimulation may assist individuals with moderate anxiety in alleviating their anxiety by enhancing HRV and lowering blood pressure.

### 4.3. Relaxation Music Group

Among participants with no anxiety, significant increases were observed in nLF and nLF/nHF, coupled with a significant decrease in nHF (*p* < 0.05; Table 2). This suggests heightened sympathetic nervous system (LF) activity and diminished parasympathetic nervous system (HF) activity [33]. This outcome indicates that participants without anxiety may have been in an active rather than a relaxed state [34]. While the study included a 5-min rest period for participants before testing, it is advisable for future research to implement a rest period of at least 10–15 min before conducting HRV measurements. This extended rest period allows the body to transition from potential states of activity to a calmer state, thereby reducing external factors' interference with HRV measurements.

Among participants with mild anxiety, a similar noteworthy increase in nLF and nLF/nHF, along with a significant decrease in nHF, were observed (Table 2). These findings contrast with previous research [35, 36], where listening to relaxation music typically led to a significant decrease in nLF/HF and a significant increase in nHF, indicating reduced anxiety levels. However, it is important to acknowledge that the results observed in this study among participants with mild anxiety may vary depending on different contexts, such as preoperative anxiety. Therefore, for future research, it is advisable to expand the sample size to further confirm the effectiveness of relaxation music interventions across diverse populations. Additionally, allowing participants to select relaxation music based on their personal preferences could be a valuable approach.

Among participants with moderate anxiety, a significant reduction in systolic blood pressure was observed (Table 2). Psychological stress can impact cardiovascular function, resulting in elevated blood pressure [37]. One study [38] revealed that university students experiencing stress exhibited a significant decrease in blood pressure after listening to classical music, suggesting that music can be beneficial in mitigating stress-related hypertension. Furthermore, research involving hospitalized myocardial infarction patients [39] indicated that listening to music could effectively lower blood pressure and reduce anxiety. In summary, relaxation music interventions have the potential to alleviate anxiety among individuals with moderate anxiety by lowering blood pressure.

### 4.4. Differences in the Changes in HRV, Brain Wave, and Blood Pressure after the Intervention

The results in Table 3 indicate that, in comparison to BBM alone, participants with moderate anxiety exhibited a significant reduction in nLF/nHF when exposed to BBM integrated with rhythmic photic stimulation. These findings suggest that individuals with moderate anxiety might be more sensitive to the intervention effects of BBM combined with rhythmic photic stimulation. Moderate anxiety may influence the activity of the autonomic nervous system, particularly the balance between the sympathetic and parasympathetic nervous systems, at a physiological level. BBM integrated with rhythmic photic stimulation may exert a more pronounced regulatory effect on this imbalance, leading to a greater variation in the nLF/nHF ratio. This implies that patients with moderate anxiety may experience more substantial physiological and psychological benefits from the intervention, resulting in more noticeable changes in HRV. However, further research is required to explore the underlying reasons for these differences and the physiological mechanisms involved.

### 4.5. Limitation

This study has several limitations. First, the participant pool was confined to the central region of Taiwan, potentially limiting the generalizability of the research findings. Second, most participants in this study had either no anxiety or mild anxiety. Future research is recommended to include a more diverse range of participants, including those with moderate and severe anxiety, to better understand the effectiveness of the three interventions in reducing anxiety levels. Third, data collection took place during the COVID-19 pandemic, which should be considered when making broader inferences based on the results. Lastly, this study employed BBM in conjunction with relaxation music, and it is noteworthy that different types of accompanying music may yield different outcomes. Despite these constraints, the findings of this study provide a valuable foundation for future research exploring the use of BBM integrated with rhythmic photic stimulation as a means of anxiety relief.

## 5. Conclusion

The results of this study indicate that participants with moderate anxiety who received BBM integrated with rhythmic photic stimulation exhibited a significant decrease in the nLF/nHF ratio in HRV compared to those in the BBM-only group. This suggests that the combination of BBM and rhythmic photic stimulation may provide additional anxiolytic effects beyond BBM alone. Further studies are needed to explore alterations in the autonomic nervous system in correlation with EEG recordings during exposure to BBM integrated with rhythmic photic stimulation, to better understand the extent and mechanisms through which these interventions affect anxiety states.

## Figures and Tables

**Table 1:** Demographic characteristics and anxiety levels of study participants (*N* = 40).

Characteristics	Total (%)	*M* ± SD
Gender		
Male	16 (40)	—
Female	24 (60)	—
Age (year)	—	31.73 ± 8.83
BMI (kg/m^2^)	—	23.33 ± 3.30
Educational level		
Primary school	3 (7.5)	—
High school	11 (27.5)	—
University degree	24 (60)	—
Postdoctoral degree	2 (5)	—
Marital status		
Single	27 (67.5)	—
Married	13 (32.5)	—
Self-perceived health status		
Very good	1 (2.5)	—
Good	9 (22.5)	—
Fair	28 (70)	—
Poor	1 (2.5)	—
Very poor	1 (2.5)	—
Exercise frequency (days/week)		
0	14 (35)	—
1–2	13 (32.5)	—
3–4	7 (17.5)	—
More than 5	5 (12.5)	—
Smoking behavior		
None	40 (100)	—
Alcohol consumption (times/week)		
0	34 (85)	—
1–3	5 (12.5)	—
4 and more	1 (2.5)	—
Professional experience (years)		
1–2	21 (52.5)	—
3–4	11 (27.5)	—
More than 5	8 (20)	—
Length of current employment (years)		
1–2	28 (70)	—
3–4	7 (17.5)	—
More than 5	5 (12.5)	—
BAI		
Total scale	—	10.45 ± 6.90
No anxiety	15 (37.5)	3.87 ± 2.64
Mild anxiety	17 (42.5)	11.12 ± 2.18
Moderate anxiety	8 (20)	21.38 ± 3.16

*M* ± SD, mean ± standard deviation; BAI, Beck Anxiety Inventory; BMI, body mass index.

**Table 2:** Differences in HRV, brain wave, and blood pressure changes before and after intervention across anxiety levels in the three intervention groups (*N* = 40).

BAI	BBM group			BBM integrated with rhythmic photic stimulation group			Relaxation music group		
	Pretest	Posttest	*p* value	Pretest	Posttest	*p* value	Pretest	Posttest	*p* value
HRV									
No anxiety									
Mean heart rate	75.00 (70.00–83.00)	77.00 (70.00–80.00)	0.900	80.00 (64.00–82.00)	79.00 (71.00–83.00)	0.637	73.00 (68.00–79.00)	75.00 (69.00–80.00)	0.782
SDNN	58.87 (42.01–84.89)	57.67 (46.01–70.4)	0.363	58.91 (36.07–74.88)	50.27 (46.02–62.25)	0.496	54.66 (43.80–86.78)	56.37 (45.93–84.44)	0.910
nLF	45.03 (28.85–62.95)	53.48 (27.81–73.46)	0.191	48.64 (34.53–60.62)	51.91 (38.41–77.31)	0.173	42.72 (30.47–60.62)	44.19 (35.27–78.36)	0.036*⁣*^*∗*^
nHF	54.97 (37.05–71.15)	46.52 (26.54–72.19)	0.191	51.36 (39.38–65.47)	48.1 (22.69–61.59)	0.173	57.28 (39.38–69.53)	55.81 (21.64–64.73)	0.036*⁣*^*∗*^
nLF/nHF	0.99 (0.41–1.8)	1.15 (0.39–2.77)	0.233	0.95 (0.53–1.54)	1.08 (0.62–3.41)	0.050	0.75 (0.44–1.54)	0.79 (0.55–3.62)	0.020*⁣*^*∗*^
Mild anxiety									
Mean heart rate	81.00 (76.00–87.50)	79.00 (74.50–85.50)	0.192	78.00 (75.00–83.50)	76.00 (72.50–82.50)	0.004*⁣*^*∗∗*^	80.00 (75.00–87.00)	81.00 (77.50–87.00)	0.604
SDNN	51.39 (37.32–62.87)	46.00 (37.46–64.58)	0.906	51.54 (42.99–58.96)	42.50 (31.72–51.19)	0.031*⁣*^*∗*^	52.82 (39.7–63.75)	48.01 (38.47–55.34)	0.246
nLF	62.79 (41.14–74.17)	46.85 (38.04–69.09)	0.192	64.58 (40.49–75.71)	61.74 (50.71–76.04)	0.554	53.28 (32.36–65.14)	59.42 (42.64–78.66)	0.025*⁣*^*∗*^
nHF	37.21 (25.83–58.86)	53.16 (30.91–61.96)	0.906	35.42 (24.29–59.51)	38.26 (23.96–49.29)	0.554	46.72 (34.86–67.65)	40.58 (21.34–57.36)	0.025*⁣*^*∗*^
nLF/nHF	1.69 (0.70–3.04)	0.88 (0.62–2.28)	0.192	1.82 (0.69–3.37)	1.61 (1.03–3.25)	0.586	1.14 (0.48–1.88)	1.46 (0.75–3.77)	0.019*⁣*^*∗*^
Moderate anxiety									
Mean heart rate	79.00 (72.50–87.00)	76.50 (71.5–81.75)	0.064	77.00 (72.25–81.00)	74.00 (70.00–75.75)	0.012*⁣*^*∗*^	71.00 (64.00–81.50)	67.50 (63.50–76.75)	0.090
SDNN	42.40 (32.65–49.23)	49.52 (37.67–51.64)	0.208	46.16 (36.56–54.00)	48.08 (41.27–53.46)	0.575	44.95 (38.95–61.13)	57.52 (39.95–72.67)	0.161
nLF	70.10 (46.70–84.07)	52.85 (38.65–71.09)	0.327	75.57 (62.13–85.76)	42.06 (35.2–79.01)	0.012*⁣*^*∗*^	60.43 (43.17–73.32)	49.78 (41.75–69.45)	0.161
nHF	29.91 (15.93–53.3)	47.15 (28.91–61.35)	0.327	24.43 (14.24–37.87)	57.95 (20.99–64.8)	0.012*⁣*^*∗*^	39.57 (26.68–56.83)	50.22 (30.55–58.25)	0.161
nLF/nHF	2.84 (0.90–5.32)	1.16 (0.64–2.40)	0.208	3.22 (1.64–6.04)	0.74 (0.54–3.83)	0.012*⁣*^*∗*^	02.00 (0.76–3.64)	1.04 (0.73–2.39)	0.161
Brain wave									
No anxiety									
*δ*	18781.50 (11520.00–45783.00)	16827.00 (6766.00–62886.00)	0.955	17589.50 (8960.00–92704.00)	12480.00 (6161.00–35775.00)	0.125	15276.00 (10900.50–41580.50)	11695.00 (6198.00–42986.00)	0.650
*θ*	15592.00 (10289.00–35527.50)	17322.00 (8348.00–28634.00)	0.427	9754.50 (8064.50–37644.00)	9600.00 (6604.00–20508.00)	0.125	15641.50 (10343.50–36484.00)	11882.00 (7483.00–23847.00)	0.112
Low *α*	8242.00 (5148.00–12122.00)	5659.00 (4330.00–8535.00)	0.460	5776.50 (3300.50–10373.50)	5400.00 (2808.00–7109.00)	0.281	7485.00 (4993.00–13096.50)	5335.00 (4067.00–7791.00)	0.047*⁣*^*∗*^
High *α*	6698.50 (4962.50–10960.00)	5357.00 (4362.00–7529.00)	0.173	7206.50 (3687.00–8773.00)	4608.00 (3558.00–5566.00)	0.053	6674.50 (4787.00–9472.50)	5021.00 (4244.00–6983.00)	0.036*⁣*^*∗*^
Low *β*	6069.00 (3652.00–8708.50)	6407.00 (5027.00–7589.00)	0.460	6305.00 (3336.50–8445.00)	3933.00 (3220.00–6191.00)	0.191	6964.50 (3394.50–8139.50)	4949.00 (3760.00–7445.00)	0.156
High *β*	10090.00 (5086.50–16832.00)	8037.00 (5295.00–11701.00)	0.691	6841.00 (4379.00–10607.00)	5707.00 (3929.00–7140.00)	0.100	10639.50 (4554.00–11993.50)	8777.00 (4473.00–11519.00)	0.865
Low *γ*	6733.00 (3162.00–9454.00)	5382.00 (2137.00–8348.00)	0.650	4950.00 (2560.50–8376.00)	3335.00 (2196.00–4736.00)	0.053	5008.00 (2493.00–8870.50)	5229.00 (1937.00–9511.00)	0.820
High *γ*	3924.50 (2546.00–5989.50)	3761.00 (1882.00–6339.00)	0.910	2530.50 (1959.00–5240.50)	2417.00 (1629.00–3284.00)	0.020*⁣*^*∗*^	2780.00 (1959.00–8208.00)	3579.00 (1278.00–6034.00)	0.532
Mild anxiety									
*δ*	15375.50 (11308.50–24309.24)	9089.00 (5361.50–18734.50)	0.055	9369.50 (7870.25–40513.50)	5933.00 (3916.00–13323.00)	0.001*⁣*^*∗∗*^	18991.00 (11618.50–37508.67)	7162.00 (4961.50–45206.98)	0.177
*θ*	12162.50 (9023.25–21826.13)	8208.00 (6565.50–14170.50)	0.006*⁣*^*∗∗*^	10169.00 (7133.50–22094.67)	7274.00 (4999.50–12062.42)	0.002*⁣*^*∗∗*^	14639.00 (9650.25–20518.11)	7910.00 (5940.00–18864.08)	0.093
Low *α*	5907.50 (4074.25–8738.09)	4317.00 (3165.00–6377.00)	0.002*⁣*^*∗∗*^	4548.50 (3483.00–7680.81)	3042.00 (2340.00–5312.08)	0.004*⁣*^*∗∗*^	6846.00 (4356.75–7923.46)	4476.00 (3143.00–7002.00)	0.163
High *α*	5757.50 (4333.75–7621.50)	3734.00 (3200.00–4923.50)	0.002*⁣*^*∗∗*^	3985.50 (3484.75–6811.02)	3134.00 (2650.00–4658.96)	0.004*⁣*^*∗∗*^	5762.50 (4243.50–7169.64)	3825.00 (3173.00–6824.96)	0.102
Low *β*	4969.50 (3526.00–6134.44)	3333.00 (2908.00–5325.50)	0.055	3788.50 (2734.75–5781.55)	2824.00 (2482.00–3595.50)	0.001*⁣*^*∗∗*^	4729.00 (3900.25–6205.75)	4045.00 (3241.00–6426.13)	0.981
High *β*	4942.50 (3456.50–10631.00)	4407.00 (2816.50–8852.56)	0.795	3656.50 (2921.75–6389.50)	3218.00 (2343.50–4807.33)	0.006*⁣*^*∗∗*^	5019.50 (3500.25–8388.68)	6720.00 (3079.50–12410.50)	0.538
Low *γ*	2845.00 (1907.25–5871.27)	3062.00 (1361.50–5722.83)	0.906	2017.50 (1731.50–3609.00)	1888.00 (1204.50–3119.31)	0.006*⁣*^*∗∗*^	2467.00 (1785.50–5278.63)	4158.00 (1273.50–6657.50)	0.332
High *γ*	2358.00 (1548.00–4074.60)	2169.00 (965.50–3961.77)	0.981	1499.50 (1374.25–2737.50)	1414.00 (852.00–2183.88)	0.004*⁣*^*∗∗*^	1870.50 (1294.50–4236.07)	3152.00 (993.50–5113.00)	0.758
Moderate anxiety									
*δ*	14907.00 (9207.88–25183.88)	30121.50 (10914.75–39290.75)	0.263	8624.75 (8363.13–14943.13)	11256.00 (5556.25–14553.50)	0.612	19570.50 (11543.38–41332.88)	29204.50 (10695.25–41191.25)	0.575
*θ*	10475.25 (10032.13–24672.38)	17195.50 (9845.25–25307.00)	0.779	10816.25 (7653.25–13388.13)	9817.00 (6442.75–11592.25)	0.208	17219.25 (10482.00–28382.25)	22656.00 (9452.00–37256.00)	0.575
Low *α*	5064.50 (3967.25–9516.13)	7320.00 (4066.75–8780.50)	0.779	5509.50 (3439.63–6913.00)	4412.50 (2954.25–6002.00)	0.123	7500.50 (4169.75–10121.63)	8821.00 (4947.25–15106.00)	0.674
High *α*	5485.25 (4116.50–10589.63)	7061.50 (3862.25–7778.25)	0.889	5282.00 (3711.25–6136.38)	4804.00 (2867.25–4839.50)	0.128	6922.25 (4404.38–9998.75)	7868.50 (4906.25–13743.75)	0.575
Low *β*	3975.50 (3128.50–8969.25)	5479.00 (3440.25–7070.50)	0.674	3782.50 (3326.25–4592.88)	4079.00 (2508.75–5858.00)	1.000	6505.25 (3707.00–8952.88)	7780.50 (4553.00–9939.75)	0.161
High *β*	4008.00 (3173.75–9977.13)	5167.50 (3512.75–9118.25)	0.779	3919.00 (3197.63–5284.63)	3523.00 (3220.25–7282.75)	0.779	8146.75 (4351.75–12178.63)	9886.50 (7092.00–13817.00)	0.123
Low *γ*	2989.25 (1897.88–6963.13)	3246.50 (1727.00–6976.50)	1.000	2173.50 (1868.00–3004.25)	2428.50 (1701.75–4128.50)	0.889	4731.75 (3088.00–7531.63)	6888.00 (4904.75–7418.00)	0.401
High *γ*	2197.25 (1395.50–4844.25)	1975.50 (1143.00–4830.50)	0.779	1513.75 (1413.13–2337.88)	1848.50 (1297.75–3249.75)	0.484	4197.50 (2615.63–6534.38)	4105.00 (3321.25–5328.00)	1.000
Blood pressure									
No anxiety									
Systolic pressure	122.00 (110.00–126.00)	117.00 (103.00–128.00)	0.209	125.00 (112.00–128.00)	122.00 (110.00–132.00)	0.432	119.00 (112.00–126)	114.00 (106.00–132.00)	0.073
Diastolic pressure	80.00 (70.00–90.00)	81.00 (75.00–92.00)	0.400	90.00 (75.00–93.00)	84.00 (73.00–92.00)	0.045*⁣*^*∗*^	84.00 (74.00–90.00)	85.00 (71.00–91.00)	0.609
Mild anxiety									
Systolic pressure	124.00 (113.00–137.00)	125.00 (118.00–143.00)	0.243	120.00 (109.50–136.00)	121.00 (109.50–133.00)	0.190	126.00 (117.00–141.00)	120.00 (104.00–140.50)	0.006*⁣*^*∗∗*^
Diastolic pressure	78.00 (71.00–88.50)	81.00 (74.50–95.50)	0.068	81.00 (73.50–90.00)	78.00 (67.50–91.00)	0.191	78.00 (70.00–93.5)	76.00 (67.00–90.00)	0.400
Moderate anxiety									
Systolic pressure	111.50 (106.50–114.00)	112.00 (103.25–118.75)	0.752	113.00 (106.50–126.75)	107.00 (105.00–116.75)	0.018*⁣*^*∗*^	117.00 (108.75–123.00)	109.00 (105.75–115.25)	0.018*⁣*^*∗*^
Diastolic pressure	72.50 (70.25–77.50)	72.00 (68.00–74.00)	0.061	76.00 (68.5–79.00)	72.50 (65.25–75.00)	0.011*⁣*^*∗*^	78.00 (72.25–84.00)	70.00 (69.00–83.25)	0.611

*p* value: Wilcoxon signed rank test, *⁣*^*∗*^*p* < 0.05, *⁣*^*∗∗*^*p* < 0.01. BAI, Beck Anxiety Inventory; BBM, binaural beat music; HRV, heart rate variability; SDNN, standard deviation of NN intervals; nLF, normalized low frequency; nHF, normalized high frequency.

**Table 3:** Differences in HRV, brain wave, and blood pressure changes among individuals with different levels of anxiety in the three intervention groups (*N* = 40).

BAI	BBM group	BBM integrated with rhythmic photic stimulation group	Relaxation music group	*p* value
HRV				
No anxiety				
Mean heart rate	−2.00 (−4.00 to 4.00)	2.00 (−4.00 to 4.00)	0.00 (−2.00 to 3.00)	0.981
SDNN	3.88 (−6.06 to 13.46)	1.91 (−13.71 to 8.21)	2.36 (−13.71 to 10.25)	0.510
nLF	4.91 (−6.43 to 14.86)	6.35 (−3.79 to 12.69)	8.82 (0.30–22.03)	0.492
nHF	−4.91 (−14.86 to 6.43)	−6.35 (−12.69 to 3.79)	−8.82 (−22.03– to 0.30)	0.492
nLF/nHF	−0.03 (−0.48 to 0.08)	0.18 (−0.07 to 1.71)	0.36 (0.01–1.71)	0.270
Mild anxiety				
Mean heart rate	−3.00 (−5.00 to 1.50)	−2.00 (−3.50 to 0.00)	−2.00 (−5.00 to 3.50)	0.570
SDNN	−3.05 (−14.33 to 17.85)	−4.14 (−11.26 to 0.72)	−5.47 (−10.80 to 7.77)	0.336
nLF	1.65 (−15.61 to 8.93)	−3.84 (−15.43 to 21.76)	10.85 (0.41–20.34)	0.731
nHF	−1.65 (−8.93 to 15.61)	3.84 (−21.76 to 15.43)	−10.85 (−20.34– to 0.41)	0.731
nLF/nHF	0.00 (−1.19 to 0.52)	−0.31 (−1.46 to 1.16)	0.63 (0.00–1.50)	0.056
Moderate anxiety				
Mean heart rate	−3.00 (−6.00 to 0.50)	−3.50 (−5.00 to −2.25)	−2.00 (−6.75 to −0.25)	0.657
SDNN	10.05 (−5.10 to 16.57)	1.97 (−8.40 to 18.53)	4.86 (−3.20 to 21.92)	0.607
nLF	−2.72 (−33.17 to 8.56)	−16.75 (−27.57 to −5.59)	−9.21 (−14.95 to 1.39)	0.882
nHF	2.72 (−8.56 to 33.17)	16.75 (5.59–27.57)	9.21 (−1.39 to 14.95)	0.882
nLF/nHF	0.07 (−0.30 to 1.03)	−1.37 (−2.47 to −0.91)	−0.38 (−1.81 to 0.28)	0.021*⁣*^*∗*^
	Post hoc: BBM integrated with rhythmic photic stimulation group > BBM group			
Brain wave				
No anxiety				
*δ*	−2174.00 (−15835.50 to 45503.50)	−4975.00 (−60696.50 to 1809.00)	−1296.00 (−9216.00 to 4601.50)	0.856
*θ*	−1660.50 (−18418.50 to 2089.50)	−493.50 (−16468.50 to 6936.50)	−1582.00 (−9658.50 to −283.50)	0.880
Low *α*	−105.50 (−5005.50 to 1551.50)	−291.50 (−2837.00 to 1117.00)	−944.00 (−3711.50 to 15.00)	0.983
High *α*	−1253.50 (−4234.50 to 852.50)	−1174.50 (−3630.00 to 344.50)	−3174.50 (−6131.00 to 292.00)	0.436
Low *β*	531.50 (−1109.00 to 1997.00)	−391.50 (−3390.50 to 794.00)	−465.50 (−1477.50 to 406.50)	0.635
High *β*	931.00 (−4388.50 to 3634.50)	−1305.00 (−3223.00 to 587.50)	−104.50 (−2429.50 to 1381.00)	0.229
Low *γ*	476.50 (−2498.00 to 3404.00)	−970.00 (−2159.50 to 348.00)	62.50 (−1214.00 to 605.50)	0.696
High *γ*	−138.00 (−1479.50 to 1689.00)	−847.00 (−2566.00 to 136.00)	−83.50 (−1521.00 to 1419.50)	0.538
Mild anxiety				
*δ*	−5999.50 (−11837.00 to −1911.50)	−1582.00(−9658.50 to −283.50)	−5651.50 (−11581.75 to 4824.50)	0.985
*θ*	−5412.00 (−8840.81 to −1917.00)	−3577.50 (−9643.50 to −1025.50)	−3521.56 (−6972.50 to 784.50)	0.731
Low *α*	−1889.06 (−3721.25 to −970.25)	−1170.50 (−3169.00 to −561.75)	−661.00 (−3416.25 to 737.75)	0.250
High *α*	−2028.50 (−2632.75 to −697.25)	−1048.00 (−3052.50 to −782.00)	−786.00 (−4287.25 to 437.00)	0.327
Low *β*	−858.00 (−2213.25 to 227.50)	−1064.00 (−2423.25 to −469.25)	220.00 (−1406.25 to 906.49)	0.391
High *β*	−141.00 (−1838.25 to 1714.50)	−655.50 (−2282.25 to −176.50)	503.13 (−1091.50 to 2814.00)	0.096
Low *γ*	−349.00 (−1495.50 to 1385.50)	−423.00 (−1415.25 to −23.00)	443.00 (−753.50 to 2380.50)	0.427
High *γ*	−69.15 (−866.75 to 579.75)	−380.50 (−1035.80 to −114.50)	−130.00 (−1539.50 to 3313.25)	0.118
Moderate anxiety				
*δ*	7125.75 (−2936.25 to 13449.88)	−836.50 (−3926.75 to 3219.00)	5226.75 (−10410.75 to 15051.88)	0.587
*θ*	1278.25 (−3786.25 to 4891.25)	−944.00 (−4267.88 to 576.00)	1711.25 (−7969.88 to 11084.63)	0.368
Low *α*	828.25 (−2053.38 to 3425.75)	−773.50 (−3500.13 to 804.38)	1085.00 (−1955.00 to 3118.25)	0.393
High*α*	75.50 (−2585.50 to 1678.88)	−769.50 (−2923.38 to −90.25)	1077.50 (−3394.38 to 4488.88)	0.417
Low*β*	326.00 (−626.75 to 1469.25)	42.75 (−1289.00 to 1110.50)	1173.50 (−1156.13 to 3726.50)	0.657
High *β*	−13.00 (−724.38 to 1756.75)	113.50 (−1139.25 to 986.13)	1291.25 (−141.75 to 4603.25)	0.607
Low *γ*	99.75 (−1100.88 to 1003.38)	−18.00 (−598.25 to 1062.75)	405.00 (−827.38 to 3545.25)	0.882
High *γ*	−75.25 (−654.25 to 897.50)	99.25 (−404.75 to 449.25)	95.75 (−2853.63 to 2613.00)	0.882
Blood pressure				
No anxiety				
Systolic pressure	−2.00 (−8.00 to 2.00)	−3.00 (−9.00 to 8.00)	−6.00 (−9.00 to −2.00)	0.624
Diastolic pressure	1.00 (−2.00 to 4.00)	−3.00 (−8.00 to 0.00)	−2.00 (−7.00 to 8.00)	0.375
Mild anxiety				
Systolic pressure	2.00 (−1.50 to 6.00)	−3.00 (−9.00 to 3.50)	−7.00 (−12.00 to 0.00)	0.057
Diastolic pressure	1.00 (−0.50 to 6.50)	−1.00 (−6.00 to 2.50)	0.00 (−3.50 to 2.50)	0.157
Moderate anxiety				
Systolic pressure	0.00 (−6.75 to 1.00)	−3.50 (−8.75 to −1.25)	−5.00 (−13.50 to −1.50)	0.223
Diastolic pressure	−2.00 (−2.75 to 0.75)	−4.00 (−5.75 to −3.25)	−2.50 (−12.75 to 3.75)	0.197

*p* value: Friedman test, after intervention minus that before intervention, *⁣*^*∗*^*p* < 0.05; Post hoc: Wilcoxon signed rank test with Bonferroni correction (*p* value <0.017 was considered significant); BAI, Beck Anxiety Inventory; BBM, binaural beats music; HRV, heart rate variability; SDNN, standard deviation of NN intervals; nLF, normalized low frequency; nHF, normalized high frequency.

## Data Availability

The datasets used and/or analyzed during the current study are available from the corresponding author on reasonable request.

## Supplementary Materials

Supplementary Materials Beck Anxiety Inventory.
