# Supplementary material for: Effects of Binaural Beat Music Integrated with Rhythmical Photic Stimulation on Anxiety Reduction among Healthy Daycare Center Staff
Source: Depress Anxiety. 2024 Jul 18;2024:5556702. doi: 10.1155/2024/5556702 (PMC11918958; doi:10.1155/2024/5556702)
Supplement: Supplementary Materials — Beck Anxiety Inventory. [file 5556702.f1.pdf]

Beck Anxiety Inventory

Below is a list of common symptoms of anxiety. Please carefully read each item in the list. Indicate how much you have been bothered by that symptom during the past month, including today, by circling the number in the corresponding space in the column next to each symptom.

	Not At All	Mildly but it didn't bother me much.	Moderately - it wasn't pleasant at times	Severely – it bothered me a lot
Numbness or tingling	0	1	2	3
Feeling hot	0	1	2	3
Wobbliness in legs	0	1	2	3
Unable to relax	0	1	2	3
Fear of worst happening	0	1	2	3
Dizzy or lightheaded	0	1	2	3
Heart pounding/racing	0	1	2	3
Unsteady	0	1	2	3
Terrified or afraid	0	1	2	3
Nervous	0	1	2	3
Feeling of choking	0	1	2	3
Hands trembling	0	1	2	3
Shaky / unsteady	0	1	2	3
Fear of losing control	0	1	2	3
Difficulty in breathing	0	1	2	3
Fear of dying	0	1	2	3
Scared	0	1	2	3
Indigestion	0	1	2	3
Faint / lightheaded	0	1	2	3
Face flushed	0	1	2	3
Hot/cold sweats	0	1	2	3
**Column Sum**				

***Scoring* -** Sum each column. Then sum the column totals to achieve a grand score. Write that score here ____________ .
